# Production of α-Tocopherol–Chitosan Nanoparticles by Membrane Emulsification

**DOI:** 10.3390/molecules27072319

**Published:** 2022-04-03

**Authors:** Sonia Trombino, Teresa Poerio, Federica Curcio, Emma Piacentini, Roberta Cassano

**Affiliations:** 1 Department of Pharmacy, Health and Nutritional Sciences, University of Calabria, 87036 Rende, Italy; sonia.trombino@unical.it (S.T.); federica.curcio@unical.it (F.C.); 2 Institute on Membrane Technology (ITM–CNR), Via Pietro BUCCI, c/o University of Calabria, Cubo 17C, 87036 Rende, Italy; t.poerio@itm.cnr.it

**Keywords:** membrane emulsification, nanoparticle production, chitosan, α-tocopherol, antioxidant property

## Abstract

α-tocopherol (α-T) has the highest biological activity with respect to the other components of vitamin E; however, conventional formulations of tocopherol often fail to provide satisfactory bioavailability due to its hydrophobic characteristics. In this work, α-tocopherol-loaded nanoparticles based on chitosan were produced by membrane emulsification (ME). A new derivative was obtained by the cross-linking reaction between α-T and chitosan (CH) to preserve its biological activity. ME was selected as a method for nanoparticle production because it is recognized as an innovative and sustainable technology for its uniform-particle production with tuned sizes and high encapsulation efficiency (EE%), and its ability to preserve the functional properties of bioactive ingredients operating in mild conditions. The reaction intermediates and the final product were characterized by ^1^HNMR, Fourier-transform infrared spectroscopy (FTIR) and differential scanning calorimetry (DSC), while the morphological and dimensional properties of the nanoparticles were analyzed using electronic scanning microscopy (SEM) and dynamic light scattering (DLS). The results demonstrated that ME has high potential for the development of α-tocopherol-loaded nanoparticles with a high degree of uniformity (PDI lower than 0.2), an EE of almost 100% and good mechanical strength, resulting in good candidates for the production of functional nanostructured materials for drug delivery. In addition, the chemical bonding between chitosan and α-tocopherol allowed the preservation of the antioxidant properties of the bioactive molecule, as demonstrated by an enhanced antioxidant property and evaluated through in vitro tests, with respect to the starting materials.

## 1. Introduction

α-tocopherol is the component of vitamin E with the highest biological activity. It is a yellow viscous liquid, insoluble in water but readily soluble in organic solvents. It has proved useful in anticancer, anti-inflammatory, and antioxidant activities [1]. However, conventional formulations of tocopherol often fail to provide satisfactory bioavailability due to its hydrophobic characteristics [2]. The encapsulation allows the increase in chemical stability of the encapsulated bioactive compounds. A common strategy for hydrophobic compounds is their encapsulation by solid lipid nanoparticles (SLNs) to promote drug penetration without damaging the skin barrier, and to increase biocompatibility, biodegradability, and high loading efficiency [3]. Natural polymers, particularly polysaccharides, have been used as drug-delivery systems for a variety of therapeutic agents. Chitosan, the second most abundant natural polysaccharide after cellulose, is a biocompatible and biodegradable polymer that is widely used in the preparation of nanoparticles (NPs). It is considered safe for human food use and approved for wound-dressing applications [4]. Chitosan is also a versatile material in terms of the forms in which it can be obtained, such as beads, membranes, coatings, fibers, sponges and nanoparticles [5,6,7]. Furthermore, its chemical modification results in the formation of several derivatives (such as thiolated chitosan, carboxylated chitosan, amphiphilic chitosan, chitosan with chelating agents, PEGylated chitosan and lactose-modified chitosan) to achieve specific goals, making it a polymer with a tremendous range of potential applications [8]. Chitosan has also been used as a matrix in polymeric nanoparticles for the encapsulation of bioactive compounds such as α-tocopherol allowing its protection and delivery in a controlled manner [9].

The encapsulation of α-T in nanoparticle systems has been achieved by a variety of methods, including emulsion or microemulsion solvent displacement, interfacial polymerization or precipitation polymerization, emulsion evaporation, and emulsion diffusion [10,11,12,13,14]. Membrane emulsification is an alternative method that is increasingly used for the preparation of pharmaceutical emulsions and solid particles at the micro and nanoscale [15,16]. The process holds many advantages over conventional emulsification methods, such as low energy input, tunable droplet size, uniform size distribution, high encapsulation/loading efficiency of active ingredients, scalability, and flexibility. All these requirements are extremely attractive for the pharmaceutical industry. The uniformity of size distribution guaranties predictable and reproducible release profiles with efficient therapeutic effects while the target size is required to achieve a controlled bio-distribution, degradation and clearance [17,18]. Process flexibility has extended the use of membrane emulsification toward the production of many types of structured particles for drug-delivery release [19,20]. The high encapsulation/loading efficiencies of active ingredients and the mild operative conditions used during the process are powerful tools with which the pharmaceutical industry can design highly efficient formulations. The scalability potential of the process permits the transfer of any preparative method from a laboratory scale to an industrial scale.

In this work, new material obtained by the cross-linking of α-tocopherol and chitosan was used to produce α-tocopherol-loaded nanoparticles by the membrane-emulsification process. Their properties, particle size, particle-size distribution, and encapsulation efficiency were compared to those that were prepared using chitosan and α-tocopherol without the cross-linking reaction. The antioxidant properties of the two different formulations were also evaluated by an in vitro study on rat-liver microsomal membranes.

## 2. Results and Discussion

**Scheme 1:**
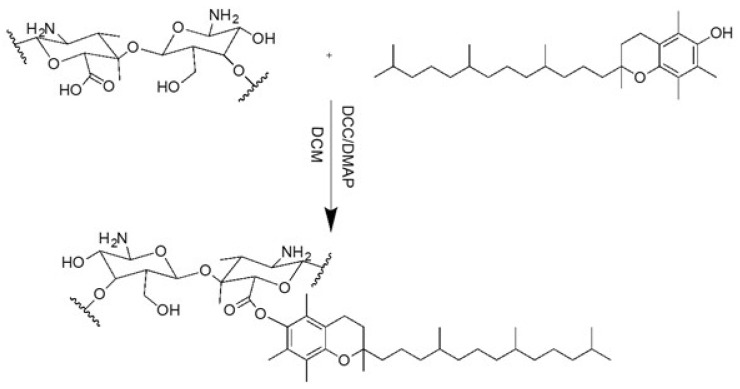
Scheme of synthetic route of CC-α-T.

To obtain carboxylated chitosan (CC), a carboxylation reaction was conducted. The esterification between CC and α-tocopherol was carried out according to the Steglich reaction by using DCC as a cross-linking agent in dry DCM at room temperature (Scheme 1).

**Figure 1:**
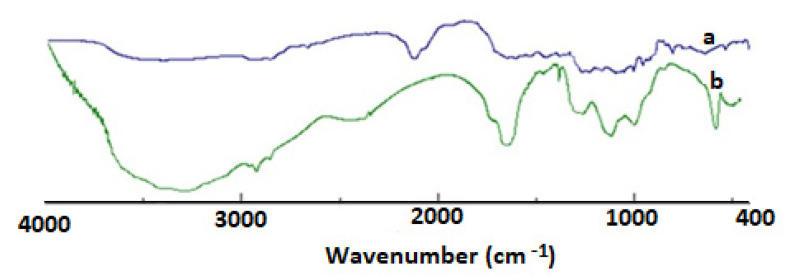
FTIR of CC (a) and CC-α-T (b).

The FTIR spectrum of CC, shown in Figure 1, reveals the presence of a new band at 1732 cm^−1^ due to the stretching vibration of the C=O of the acid, while the FTIR spectrum of CC-α shows the presence of a new band at 1772 cm^−1^ attributable to the stretching vibration of the C=O of the ester. Quantitative analysis of the ester groups was carried out by volumetric analysis to determine the degree of substitution (DS), which was found to be 0.820.

**Figure 2:**
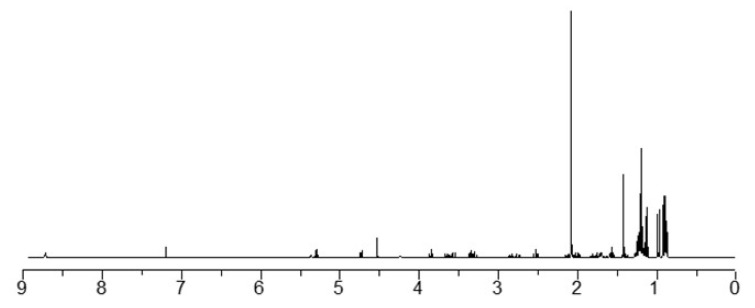
^1^H-NMR spectrum of CC-α-T.

**Figure 3:**
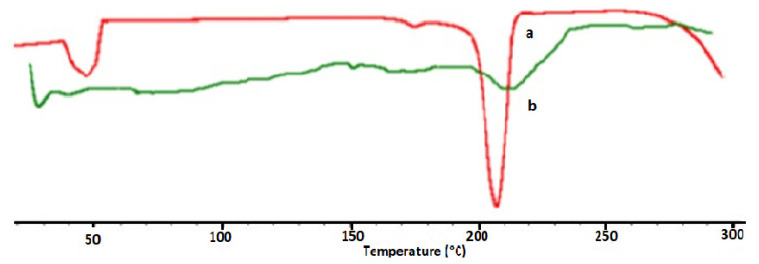
DSC curves of CC (a) and CC-α-T (b).

The ^1^H-NMR analysis provided a very broad and complex spectrum in CDCl3 in which the signals of the different CH_3_ of the alkyl chain present in the α-T (0.8–2.1 ppm) are visible. In general, the spectrum shows a rather complex zone, between 0.8 ppm and 5 ppm, containing the CH_2_ and CH signals of both chitosan and α-T (Figure 2). The DSC curves of the CC and the product CC-α-T ester are shown in Figure 3. The CC shows an endothermic peak at 207 °C (curve a) and the ester at 212 °C (curve b).

**Figure 4:**
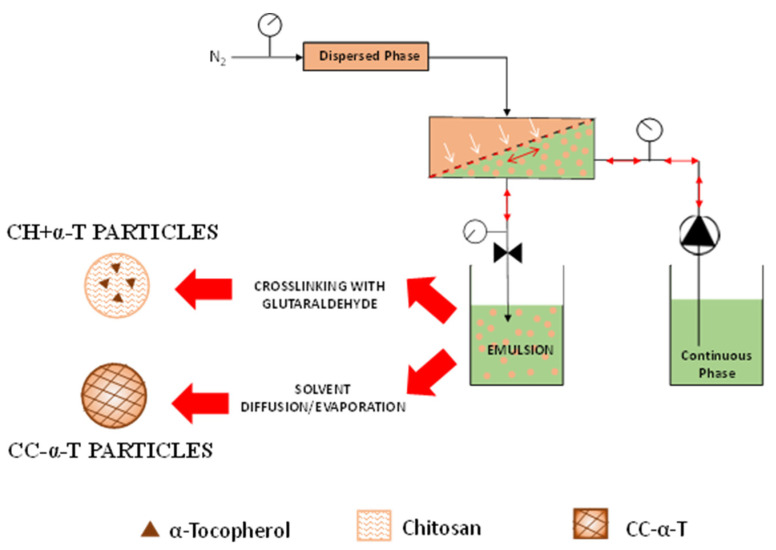
Membrane-emulsification plant used to produce W/O and O/W emulsions and solid particles obtained by cross-linking reaction (CH+α-T particles) and solvent diffusion/evaporation (CC-α-T particles).

The derivative and the unmodified chitosan were used for the encapsulation of α-tocopherol in nanoparticles produced by membrane emulsification (Figure 4). In particular, a W/O emulsion was produced by using chitosan and α-tocopherol (CH+α-T) as the dispersed phase, and the cross-linking reaction with glutaraldehyde (GA) permitted the yield of solidified particles. Alternatively, an O/W emulsion was obtained by using the cross-linked CC-α-T as the dispersed phase, and the solidified particles were obtained after solvent diffusion/evaporation

**Figure 5:**
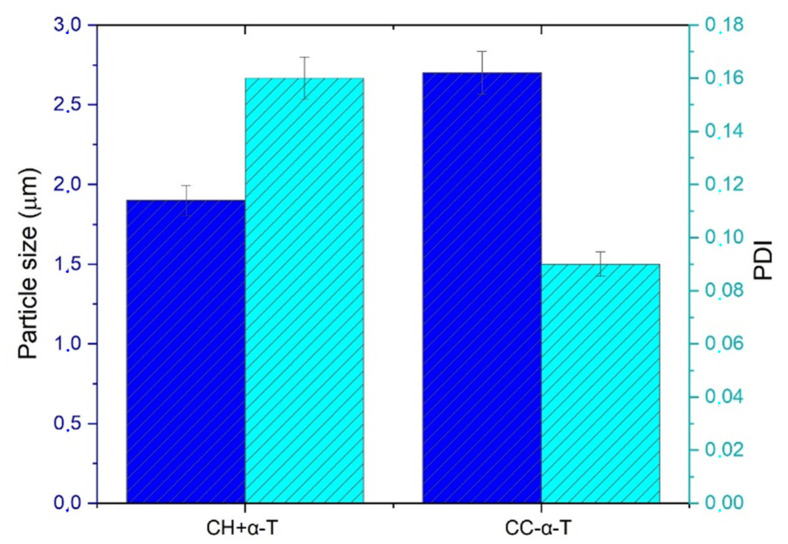
Particle size and PDI of particles produced by membrane emulsification (ME).

The membrane-emulsification process permitted the yield of very uniform CH+α-T and CC-α-T particles as indicated by dynamic-light-scattering (DLS) measurements. CH+α-T particles with sizes of 1.9 μm were obtained, which is about five times the pore size of the membrane used for emulsion production, as well as a polydispersity index (PDI), which is a measure of the heterogeneity of a sample based on size, of 0.16 (Figure 5). A slight increase in particle size (2.7 μm) was observed for the CC-α-T particles, although high uniformity was maintained (PDI = 0.09) (Figure 5).

**Figure 6:**
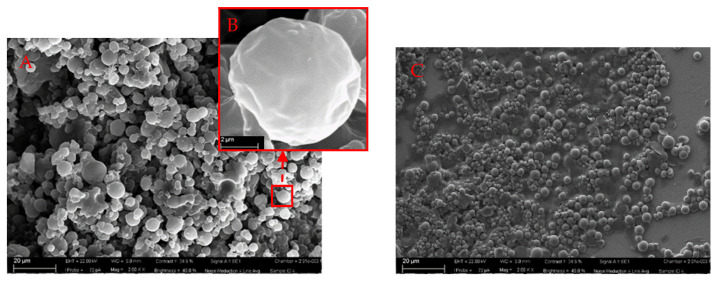
SEM micrographs: (**A**) CC-α-T SLNs at magnification of 2000× and (**B**) 18,000×; (**C**) CH+α-T particles at magnification of 2000×.

SEM images of CH+α-T and CC-α-T particles prepared by the membrane-emulsification technique are shown in Figure 6, confirming the DLS measurements. Additionally, CC-α-T particles maintained a spherical shape and a smooth surface after the solidification step during solvent diffusion/evaporation (Figure 6 A,B). Solidified CH+α-T particles were successfully produced by combining membrane emulsification and the cross-linking reaction as demonstrated in previous work (Figure 6 C) [21,22,23,24,25].

The results indicate that the membrane-emulsification technology allowed the synthesis of well-structured particles, independently of the initial formulation, composition and solvent-diffusion/evaporation method, and was suitable for the solidification of CC-α-T particles.

When α-tocopherol was added to chitosan without cross-linking (CH+α-T), the encapsulation efficiency was very low (19%) due to its diffusion from the particle core towards the external organic phase of the formulation. The cross-linking reaction (CC-α-T) guaranteed a stable interaction among the carrier material and the functional ingredient during the preparation of particles while permitting an encapsulation efficiency of 100%. The total encapsulation of α-tocopherol is due to chemical binding to CC, thereby preserving it from release while maintaining its biological activity.

**Figure 7:**
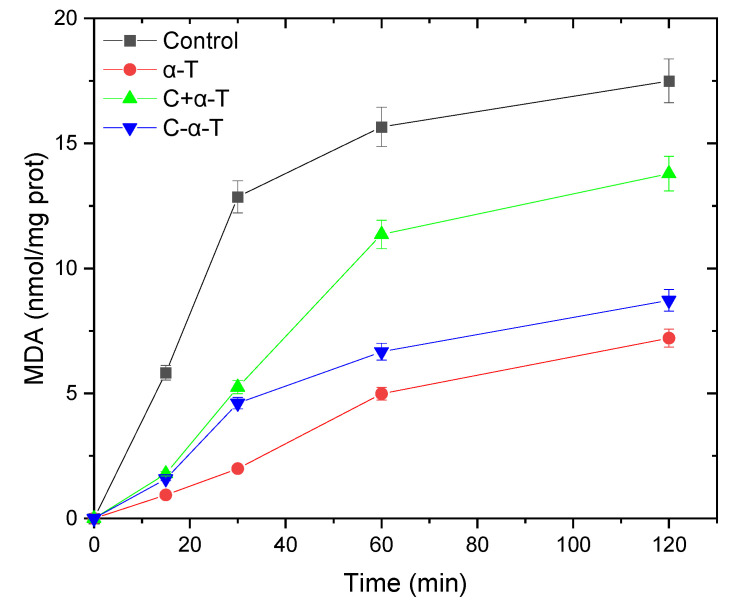
Antioxidant activity of α-T, and of CH+α-T and CC-α-T nanoparticles. Results indicate means of three independent experiments performed in triplicate (bars, standard deviation).

The ability of CC-α-T-based nanoparticles to inhibit lipid peroxidation, induced by *tert*-BOOH, was examined in rat-liver microsomal membranes over 120 min of incubation (Figure 7). The antioxidant capacity of α-T, CH+α-T and CC-α-T nanoparticles was also evaluated under the same conditions. The results indicated that all the bioactive materials exhibited antioxidant capacity by effectively protecting microsomes from *tert*-BOOH-induced lipid peroxidation. However, a lower antioxidant activity was obtained when the functional materials were simply mixed with the chitosan polymer, while the nanoparticles that showed the closest antioxidant capacity to α-T were the CC-α-T nanoparticles, confirming that chemical bonding allowed the preservation of the antioxidant capacity of the bioactive molecules. It is noteworthy that the antioxidant properties of α-tocopherol have been proposed to play a beneficial chemopreventive role against various cancers. This behavior can be understood because the induction of cell death by cytotoxic therapies normally contains an oxidative-stress component that can be decreased by antioxidants. Studies are underway to evaluate the anticancer activity of the prepared materials.

## 3. Materials and Methods

### 3.1. Materials

The reagents used for the synthesis of CC were: medium-molecular-weight chitosan, dimethylaminopyridine (DMAP), phosphate-buffered saline (PBS), dicyclohexylcarbodiimide (DCC), hydrochloric acid (HCl), sodium bile salt, butan-1-ol, tween20, NaOH, phenolphthalein, methyl red purchased from Sigma Aldrich (Milan, Italy), 85% orthophosphoric acid (H_3_PO_4_), sodium nitrite (NaNO_2_), and 85% formic acid purchased from Carlo Erba Reagenti (Milan, Italy). Dichloromethane, chloroform deuterate, diethyl ether, ethanol, methanol, acetone, n-hexane, and diethyl ether were purchased from VwR Chemicals Prolabo, Fluka Chemika-Biochemika and Lab Scan Analytical Sciences (Poland). Methylene blue was purchased from BDH Analytical Chemicals.

The polymer used in this study was chitosan (CH, average MW 327 kDa, deacetylation ≥ 75%, Sigma-Aldrich, Milan, Italy). α-tocopherol supplied by Sigma Life Science (Milan, Italy) was used as the functional biomolecule and cyclosporine supplied by Pharmalabor SRL (Canosa di Puglia, Italy). Span 80 and Pluronic F127 (Sigma-Aldrich, Milan, Italy) were used as stabilizers in the external phase during the microencapsulation process and iso-octane and ethyl acetate (Sigma-Aldrich, Milan, Italy) were used as organic solvents. Toluene and glutaraldehyde (GA) were used for the microparticle-solidification step and were purchased from Sigma-Aldrich (Milan, Italy).

### 3.2. Instruments

^1^H-NMR spectra were performed using a Bruker VM 30 spectrophotometer. FTIR spectra were recorded by a Jasco 4200 spectrophotometer using potassium bromide (KBr) foils (or powder) provided by Sigma-Aldrich (Saint Louis, MO, USA). UV–Vis spectra were recorded by Jasco V-530 UV/Vis spectrophotometer using 1 cm-thick quartz cells. Differential scanning calorimetry (DSC) was performed with the NETZSCH DSC 200 PC instrument. Samples were freeze-dried by “freezing–drying” Micro Modulyo, Edwards. Particles were analyzed by dynamic light-scattering (ZetaSizeNanoZS, Malvern Instrument) and the Z-average diameter (Z-Average) and the polydispersity index (PDI) were obtained from the autocorrelation function. Particles were also observed by an EVO MA10 Zeiss scanning electron microscope (SEM).

### 3.3. Carboxy-Chitosan Synthesis (CC)

Prior to the binding between chitosan (C) and α-tocopherol, a chitosan carboxylation reaction was performed [26]. In a three-neck flask equipped with a reflux condenser and magnetic stirrer, thoroughly flamed under nitrogen current, 1 g of chitosan (medium PM) and 40 mL of 85 wt % H_3_PO_4_ were added. After 1 h, one third of the total amount of sodium nitrite (0.043 mol) was added, keeping the solution under vigorous magnetic stirring for 5 min and then letting it stand for about 1.5 h. Next, another two portions of sodium nitrite (0.043 mol) were added to the system at the same conditions at two different moments. After 45 min, 10 mL of formic acid was added to neutralize excess sodium nitrite. The product was then precipitated with 400 mL of cold ethyl ether and 100 mL of acetone under stirring for 30 min. The mixture was then filtered and washed with distilled water and 96% ethanol. The other washes were carried out with diethyl ether and methanol until a whitish compound was obtained, which was dried under vacuum and subsequently characterized by FTIR. The sample for carboxyl-group analysis was prepared by suspending 0.05 g of carboxychitosan in a solution consisting of 2.5 mL of phosphate buffer (pH = 8) and 2.5 mL of an aqueous methylene-blue solution. The mixture was filtered and from the filtered residue, 1 mL of solution was taken, acidified with 1 mL of 0.1 N HCl and 8 mL of distilled water was added [26]. The methylene-blue content of this solution was determined using a UV-Vis spectrophotometer using a calibration line relative to methylene blue (ε = 86,126 mol^−1^·L·m ^−1^). The method is based on the binding of the free cation of methylene blue to carboxyl groups and the subsequent determination of the decrease in its concentration in the solution. The amount of free methylene blue is calculated and used in the following formula to obtain the content of carboxyl groups:

mmol COOH/g dry sample = ((7.5 − A) × 0.00313))/E(1)

where A is the total amount of free methylene blue in milligrams and the E is the weight of the dry sample (CC) in grams.

### 3.4. Reaction between α-Tocopherol and CC

The Mitsunobu reaction was used to obtain esters with high yields from a primary or secondary alcohol and a carboxylic acid using a trisubstituted phosphine and a disubstituted dazodicarboxylate [26]. Carboxychitosan and α-tocopherol were suspended in DMAc and LiCl and were kept under cold stirring for 60 min. 1,1′-(Azodicarbonyl)dipiperidine (ADDP), tributylphosphine and carboxychitosan were rapidly added. The amounts of the reagents and washing solvent are reported in Table 1. The reaction was carried out at room temperature for 24 h under a nitrogen atmosphere. The product obtained (white powder) was washed with hot methanol, filtered, dried under vacuum, and characterized by FTIR, DSC and ^1^HNMR.

To calculate the degree of substitution (DS), carboxychitosan functionalized with α-tocopherol was subjected to basic hydrolysis [26]. A quantity of 0.05 g of product was dissolved in 0.25 M NaOH ethanolic solution (Table 2). The mixture was kept under magnetic stirring at 100 °C for 17 h. The mixture was then titrated with 0.1 N HCl using phenolphthalein as the pH indicator for the first equivalence point and methyl red for the second. DS was determined according to the following equation:

DS = (PM glucosidic unit)/(g sample/n free ester) − PM free ester − PM H_2_O) (2)

where PM glucosidic unit is the molecular weight of one unit of glucose, n free ester is the number of moles of hydrolyzed ester (V2 p. eq.–V1 p. eq.), g sample is the weight of the sample (0.05 g), PM free ester is the molecular weight of the free ester, PM H_2_O is the molecular weight of water molecule (18 g/mol).

The solubility of CC-α-T was also studied in various solvents to identify the best conditions for nanoparticle production. It was found that CC-α-T is soluble in ethyl acetate and insoluble in water and acetic acid.

### 3.5. Production of α-Tocopherol-Loaded Nanoparticles Based on Chitosan by Membrane Emulsification

Membrane emulsification was used for emulsion production and the solidified particles were obtained by the subsequent secondary reaction.

For the W/O emulsion, the dispersed phase was obtained by adding 3 mL of ethanol containing α-tocopherol (10 g/L) to 27 mL of acidic solution (CH_3_COOH) of 1% *w/w* chitosan (CH). The continuous phase was a 2% *w/w* solution of Span 80 in iso-octane. A hydrophobic SPG (shirasu porous glass) membrane with a pore size of 0.4 microns was used for these experiments.

For the O/W emulsion, the dispersed phase was obtained by adding 300 µL of CC-α-T to 30 mL of ethyl acetate. The continuous phase was 2% *w/w* solution of Pluronic F127 in water saturated in ethyl acetate. A hydrophilic SPG membrane with a pore size of 0.4 microns was used for these experiments.

The membrane-emulsification experiments were carried out in a laboratory-scale system, as shown in Figure 4, consisting of the membrane module, a peristaltic pump to supply the continuous phase, and a nitrogen cylinder to feed the dispersed phase through the membrane pores. A pressure gauge located at the outlet of the cylinder allowed monitoring of the applied pressure.

The dispersed-phase circuit was filled with 30 mL of polymeric solution and the flux (*J_d_*) was determined by the volumetric flow (*Q_d_*), measuring the dispersed-phase consumption from the graduated feed cylinder due to the dispersed-phase passage through the membrane pores, according to the following equation:

$$J_{d}=\frac{Q_{d}}{A}$$

where *A* is the effective membrane area.

The continuous-phase circuit was filled with 20 mL of emulsifier solution. The pump feeding the continuous phase generates a pulsed flow. Specifically, the pump was programmed to push 2.8 mL of continuous phase at a flow rate of 850 mL/min tangentially to the membrane in one direction and in the opposite direction (“back and forth”), thus exerting the shear stress that promotes the detachment of droplets formed at the membrane pores, calculated according to the following equation:

$$\tau_{\max}=2\;a\;{\left(\pi \;f\right)}^{\frac{3}{2}}{\left(\mu_{c\;}\rho_{c}\;\right)}^{\frac{1}{2}}$$

where a is the amplitude, f is the frequency, μ_c_ is the continuous-phase viscosity and ρ_c_ is the continuous-phase density.

Emulsification was stopped when the percentage of the dispersed phase in emulsion was 10% *v*/*v*.

The W/O emulsion was used to produce CH+α-T particles by the cross-linking reaction carried out by adding toluene saturated with glutaraldehyde (cross-linking agent) under stirring for 2 h.

The O/W emulsion was used to produce CC-α-T particles by solvent diffusion/evaporation through the addition of a certain volume of no saturated continuous phase under stirring for 3 h.

The resulting CH+α-T and CC-α-T particles were centrifuged at 3500 rpm, 25 °C, for 10 min to separate the supernatant (stored for further analysis) from the pellet (subjected to repeated washing cycles with distilled water and left to dry for SEM analysis).

Particles were analyzed by dynamic light scattering), and the Z-average diameter (Z-Average) and the polydispersity index (PDI) were obtained from the autocorrelation function. Particles were also observed by scanning electron microscope (SEM).

The α-tocopherol-encapsulation efficiency (EE) in CH+α-T and CC-α-T particles was obtained according to the following equation:

$$\mathrm{EE}(\%)=\frac{\alpha T_{enc}}{\alpha T_{i}}\times 100$$

where *αT_enc_* is the total amount of α-tocopherol encapsulated in the particles and *αT_i_* the total quantity of drug initially used for the preparation.

The particles were separated from the liquid by centrifugation as previously described, and α-T content in the supernatant was determined by spectrophotometric analysis (290 nm). A calibration curve was constructed by plotting the absorbance against the corresponding concentration of α-tocopherol samples at five different concentrations (10–100 µg/mL).

### 3.6. Evaluation of Antioxidant Capacity

The antioxidant capacity was examined in rat-liver microsomal membranes, which are the ideal substrate for the lipid-peroxidation process. A total of 1 mL of microsomal suspension (0.5 mg protein) was added to an ethanolic solution consisting of 3 mL of 0.5% trichloroacetic acid (TCA), 0.5 mL of thiobarbituric acid (TBA), and 0.07 mL of 0.2% hydroxytoluenebutylate (BHT). The samples were then incubated with the microsomal suspension in a thermostatic bath at 37 °C for 24 h. At specific time intervals (0 min, 15 min, 30 min, 1 h and 2 h) the samples were withdrawn, heated at 90 °C and then centrifugated. Subsequently, the thiobarbituric-acid–malondialdehyde complex (pink color chromogen) was spectrophotometrically detected at λ = 535 nm [27,28,29,30].

## Figures and Tables

**Table 1:** Reagents and washing solvents of the synthesis of *α-tocopherol and CC*.

Reagents						Washing Solvent
CC (g)	α-tocopherol (g)	DMAc (mL)	LiCl (g)	ADDP (mL)	Bu_3_P (mL)	Metanol (mL)
1	2.54	370	3.73	2.91	3.92	358

**Table 2:** Reagents used in the volumetric analysis of substitution degree.

Reagents			
CC-α-T (g)	NaOH (etanol.) (mL)	HCl 1 p. eq. (mL	HCl 2 p. eq. (mL)
0.05	5	0.6	1

## Data Availability

The data presented in this study are available in article.

## Sample Availability

Samples of the compounds are available from the authors.
